# CMTM6, the newly identified PD-L1 regulator, correlates with PD-L1 expression in lung cancers

**DOI:** 10.1016/j.bbrep.2019.100690

**Published:** 2019-10-03

**Authors:** Feng Gao, Jing Chen, Jia Wang, Peixiang Li, Sheng Wu, Jue Wang, Yong Ji

**Affiliations:** a Department of Pathology, Jingjiang People's Hospital, Jingjiang, Jiangsu, 214500, China; b Department of Neurology, The Fourth Affiliated Hospital of Jiangsu University, Zhenjiang, Jiangsu, 212001, China

**Keywords:** CMTM6, PD-L1, Immunohistochemistry, Lung carcinomas, Therapy

## Abstract

Modulation of Immune check point regulators, especially the PD-1/PD-L1 axis, plays a critical role in successful management of a small proportion of lung cancer patients, but not so effective in the rest of lung cancer patients. A better understanding of immunotherapy non-responsive or resistant patients therefore warranted for future development of novel therapeutics. The newly identified regulator CMTM6 (CKLF-like MARVEL transmembrane domain containing 6) has been reported to serves as the stabilizer of PD-L1 and enhances the inhibitory effect of PD-L1 on immune system in both cell line and animal models, but its clinical relevance associated with PD-L1 is unknown and the current study is designed to address this question. The study using immunohistochemistry demonstrated that CMTM6 positivity from 15 out of 19 types of cancers with our in-house tissue microarray, and PD-L1 expression is always found only in CMTM6 positive cancers. CMTM6 and PD-L1 expression were analyzed in 81 lung cancer patient sample, and we observed that CMTM6 expression correlated with cancer histotypes and inversely correlated with cancer metastases, but not with patients’ age and gender. No PD-L1 expression was observed in negative CMTM6 samples. Higher expression PD-L1 is also associated with higher CMTM6 expression. In summary, CMTM6 expression is associated with PD-L1 expression, as well as lung cancer histotypes and metastasis. The results thus for the first time confirmed earlier reports on CMTM6/PD-L1 connection, from a clinical aspect of analysis.

## Highlights

• Confirmed the CMTM6 expression in different cancer tissues include lung cancers.
• Addressed the correlation of CMTM6 and PD-L1 expression in clinical samples.
• Demonstrated the correlation between CMTM6 expression and lung cancer histotypes.

## 1 Introduction

The identification of the Programmed Cell Death – 1/Ligand 1 (PD-1/PD-L1) immune checkpoint pathway has shown great promise as a therapeutic target to elicit immune response against cancer cells [1]. The PD-1 protein is a receptor expressed on the surface of T cells, B cells and macrophages that delivers co-inhibitory signal, playing a critical role in maintaining peripheral tolerance and preventing autoimmunity [2,3]. This co-inhibitory signal, however, is delivered only when PD-L1 is bound to PD-1. As an important ligand of PD-1, PD-L1 expresses on the cell surface of antigen presenting cells, including tumor cells and tumor infiltration lymphocytes (TILs). Binding of PD-L1 to PD-1 results in the induction of apoptosis of antigen specific T cells, while reduction of apoptosis of regulatory T cells [4,5]. Though being essential under physiological condition to prevent autoimmunity, PD-1/PD-L1 pathway is in the same way exploited by cancer cells with increased PD-L1 expression to inhibit the immune response and thus to evade successfully host immune surveillance.

Current therapeutic applications take advantage use of immune checkpoint inhibitors that target the PD-1/PD-L1 pathway. However, the blockade of PD-1/PD-L1 for therapeutic again seems only work on a small sub-group of patients, other patients either show no response or develop resistance after initial response [1]. The solution to this challenge will require a better understanding of the mechanisms and context of the PD-1/PD-L1 pathway as well as the regulation of PD-L1 expression from other co-factors. Recent identification of an important regulator of PD-L1, CKLF-like MARVEL transmembrane domain containing protein 6 (CMTM6), offers such a glimpse of hope in this direction for designing effective immunotherapy in resistant patients [6,7].

CMTM6, together with other 7 members from the chemokine-like factor superfamily (CKLF), was identified as early as 2003 [8], but its biological function was unknown until two independent reports published on Nature in late 2017 [6,7]. It was reported that CMTM6/CMTM4 binds to PD-L1 and stabilizes PD-L1, resulting in enhanced inhibitory impact on immune system and successful escape of immune surveillance [6,7]. CMTM6 plays the major/dominant role in this process, while CMTM4 serves as backup and functions only when CMTM6 expression is not present. This provides the basic principle that a successful inhibition of the binding of CMTM6 with PD-L1 may recover existing suppressed immune response and serve as a novel potential therapeutic target. But both studies were reported at genetic and cellular levels with different cell lines. The exact role of CMTM6 and especially its correlation with PD-L1 in human clinical samples has been so far unclear.

To study the regulation of PD-L1 through CMTM6 in clinical samples, we analyzed both PD-L1 and CMTM6 expression in the same set of cancer samples by immunohistochemistry and have demonstrated the expression of CMTM6 and PD-L1 in 15 and 8 out of 19 types of cancers, respectively. Moreover, we observed that all PD-L1 positive cancers show positivity of CMTM6. Furthermore, we for the first time reported that CMTM6 express in a cohort of lung cancers, and it is associated with lung cancer histotypes, metastases. Consistent with previous finding, CMTM6 expression is not only correlated with PD-L1 expression, but could also be a requirement for PD-L1 expression.

## 2 Materials and methods

### 2.1 Ethics statement on human tumor samples

Included were 81 lung cancer tissues collected from patients who underwent surgery at Jingjiang People's Hospital. All patients received surgery and the following systematic chemotherapy. Immunotherapy was not applied to any of the patients in this study. Patient diagnosis was concluded after histological evaluation by pathologists of the hospital. Consent documents and study protocols were approved by Jingjiang People's Hospital Medical Ethics Board, and were in compliance with the Declaration of Helsinki, 1975.

### 2.2 Immunohistochemistry (IHC) staining of CMTM6 and PD-L1 in tissue microarray (TMA)

An in-house tissue microarray was constructed composed of 42 samples from 19 types of cancers randomly chosen from hospital patient sample (Table 1). The CMTM6 (Clone ACH0275) and PD-L1 (Clone AHC0271) monoclonal antibodies used in this study are purchased from Applied Biological Materials Inc. (BC, CANADA). IHC was performed with CMTM6 and PD-L1 antibodies using the Leica BondMax automatic IHC staining machine, following the condition described briefly below. TMA slides were firstly baked at 60 °C before the antigen retrieval was conducted with Leica Epitope Retrieval Solution 2 (pH = 9) at 99 °C for 30 min. CMTM6 and PD-L1 primary antibodies were diluted to 1.0 μg/ml and 4.0 μg/ml, respectively, and the incubation was set at ambient temperature for 30 min. Then signal was detected with Leica Bond Polymer Refine Detection kit (Leica Biosystems, ON Canada). Post-primary antibody incubation and polymer incubation were set at 8 min for both, respectively, and followed by Haematoxylin counter staining for 5 min at room temperature. Staining result was observed and recorded with Leica DM1000 microscope (Leica Microsystems, ON, Canada).

**Table 1:** TMA composition and CMTM6/PD-L1 expression.

Sample	Bladder 1	Bladder 2	Brain 1	Brain 2	Brain 3	Breast 1	Breast 2
PD-L1	P	N	N	N	N	N	N
CMTM6	P	N	N	N	N	N	P
Sample	Cervix 1	Cervix 2	Cervix 3	Cervix 4	Cervix 5	Colon 1	endometrium
PD-L1	P	N	N	N	N	N	N
CMTM6	P	P	P	P	N	P	P
Sample	Endometrium 2	Esophagus 1	Esophagus 2	Gallbladder 1	Intestine1	Kidney 1	Kidney 2
PD-L1	N	N	N	N	N	N	N
CMTM6	N	N	N	P	N	N	P
Sample	Liver 1	Liver 2	Liver 3	Liver 4	Liver 5	Lung 1	Lung 2
PD-L1	N	P	N	N	N	P	P
CMTM6	P	P	N	P	P	P	P
Sample	Melanoma 1	Ovary 1	Ovary 2	Ovary 3	Ovary 4	Pancreas 1	Pancreas 2
PD-L1	P	P	P	P	P	P	N
CMTM6	P	P	P	P	P	P	P
Sample	Rectum 1	Rectum 2	Stomach 1	Stomach 2	Throat 1	Thyroid 1	Thyroid 2
PD-L1	N	N	P	N	N	N	N
CMTM6	N	P	P	P	N	P	P

**Note:** P = positive, N=Negative.

### 2.3 Patient selection

Formalin-Fixed Paraffin-Embedded (FFPE) samples from 81 patients previously diagnosed with lung cancers were collected and tested in this study. The samples recovered were from 31 female and 50 male patients with a median age of 63 years old (range of 33–78 years). We selected the patients that all have undergone surgery and similar following chemotherapy treatment to avoid the possible sampling bias. The samples were pre-classified as one of the four different histotypes, lung carcinomas in situ (CIS), lung squamous cell carcinomas (LSCC), lung adenocarcinomas (LAC) and small cell lung cancer (SCLC), by licensed pathologists at the Pathology Department of the Hospital, and among these samples, 12 samples showed metastasis to lymph nodes, 51 samples did not, and the other 18 samples unknown. The clinicopathological information was summarized in Table 2.

**Table 2:** Clinical pathological characteristics of patients and its correlation with CMTM6 expression in lung cancers.

Variables	Expression levels (n = 81)				P-value (p = 0.05)
	0	1	2	3	
**Histotypes**					P_ALL*_<0.0001 P_NS/S**_<0.0001
CIS	2	1	17	3	
LSCC	0	9	11	1	
LAC	0	3	9	9	
SCLC	12	2	2	0	
**Metastasis to lymph node**					P = 0.017
LN+	1	6	4	1	
LN-	2	6	33	10	
uncertain	11	3	2	2	
**Age**					
<63	7	5	23	4	P = 0.149
≥63	7	10	16	9	
**Gender**					P = 0.084
Female (F)	4	2	19	6	
Male (M)	10	13	20	7	

* P_ALL*_<0.0001: all 4 different types of lung cancers were analyzed individually.

**P_NS/S**_<0.0001: samples were analyzed with grouping CIS, LSCC and LAC together as NSCLC and compared with the SCLC group.

### 2.4 IHC staining with lung cancer samples

All the 81 lung cancer samples were fixed in 10% Neutral Buffered Formalin, embedded and serial sectioned to thickness of 4 μm and attached to a positively charged glass slide. IHC staining was performed with CMTM6 and PD-L1 primary antibodies at 1.0 μg/ml and 4.0 μg/ml, respectively, following the protocol described above. CMTM6 and PD-L1 expression were both evaluated by three individual pathologists who were blinded to the experimental groups. The evaluation of CMTM6 and PD-L1 expression was both determined from tumor cells and immune cells, and scored into 4 different categories (0–3), respectively.

### 2.5 Statistical analysis

The PD-L1 and CMTM6 staining score were collected as described above, and analyzed directly against the clinical pathological parameters in addition to a thorough evaluation of any correlation among them. A Chi-square test was to determine the significance of the difference of each data with p-values <0.05 were considered significant (Table 2, Table 3).

**Table 3:** Correlation of CMTM6 and PD-L1 expression.

CMTM6	PD-L1					P value
	0	1	2	3	Total	
0	14	0	0	0	14	0.001
1	11	3	0	1	15	
2	37	2	0	0	39	
3	8	1	0	4	13	
Total	70	6	0	5	81	

## 3 Results

### 3.1 CMTM6 and PD-L1 expression in different cancers on TMA

To evaluate CMTM6 and PD-L1 expression in different types of cancers, IHC was performed with a TMA composed 42 samples from 19 types of cancers. CMTM6 positivity was detected with 28 samples (66.7%) from 15 types of cancers, but negative with other 4 types of cancers. PD-L1 expression was present with 12 samples (28.5%) from 8 types of cancers, while negative with the other 11 types of cancers (Table 1), PD-L1 positive samples are all positive with CMTM6 expression, none of CMTM6 negative samples have positivity for PD-L1 expression.

**Fig. 1:**
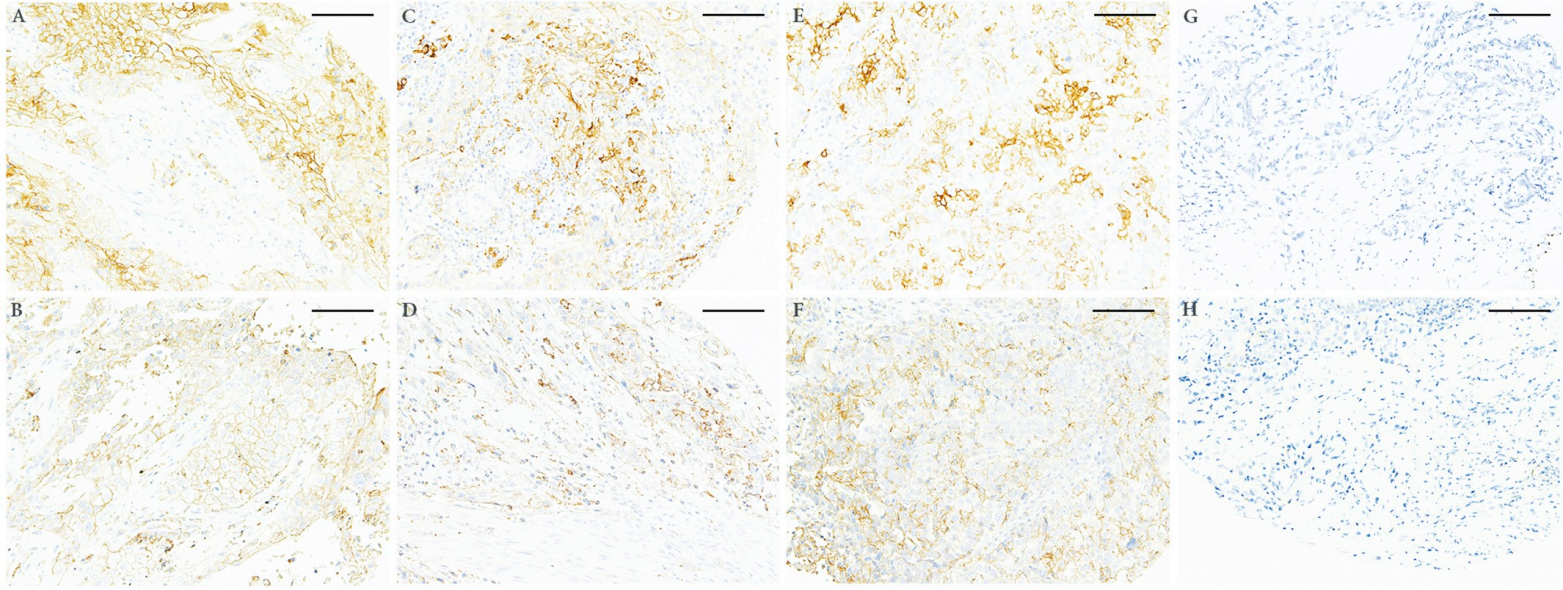
**PD-L1 and CMTM6 expression in different types of cancers in TMA**. IHC staining of PD-L1 and CMTM6 was performed with paired TMA slides and representative illustration was shown. A, C, E and G are PD-L1 staining and B, D, F and H are CMTM6 staining, with lung cancer (A and B), bladder cancer (C and D), Ovary cancer (E and F) and prostate cancer (G and H), respectively. We observed clear membranous positivity in panel A–F and negative staining in panel G and H. Magnification is 400× and Scale bar = 50  μm.

We observed positive staining of CMTM6 and PD-L1 from both tumor cells and immune cells, with the feature of typical membranous staining. Representative CMTM6 and PD-L1 staining were shown in Fig. 1.

### 3.2 CMTM6 and PD-L1 expression in lung cancers

**Fig. 2:**
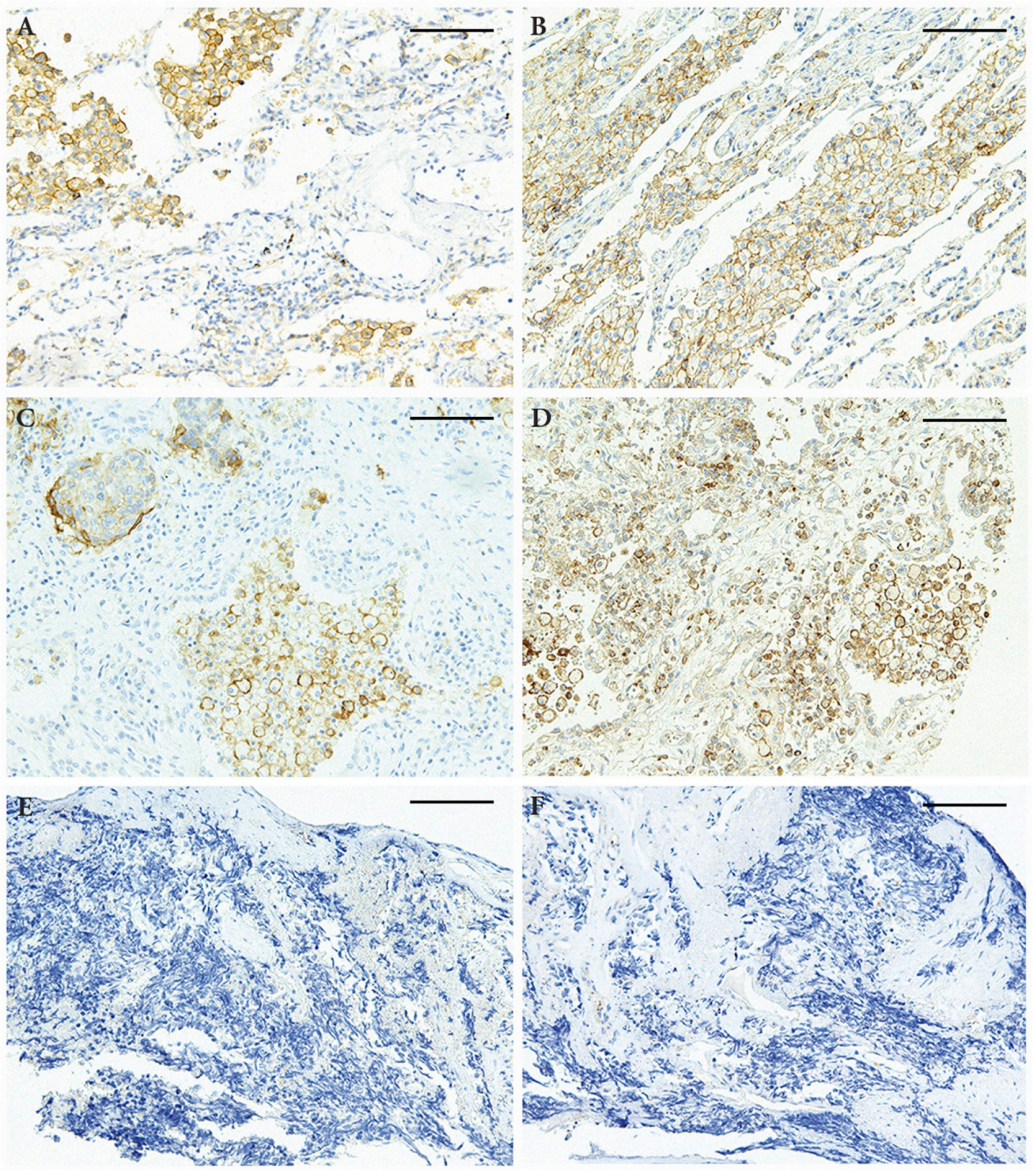
**PD-L1 and CMTM6 expression in lung cancer samples**. PD-L1 (A, C, E) and CMTM6 (B, D, F) expression are correlated in paired lung cancer tissues tested in this study. A and B are representative strong PD-L1 and CMTM6 staining in one NSCLC sample, C and D are weak PD-L1 and CMTM6 staining in another NSCLC sample, and E and F are negative staining in one SCLC sample. Magnification is 400× and Scale bar = 50  μm.

After the observation of the expression of CMTM6 and PD-L1 in different types of cancers, we focused the rest of our study on lung cancers as PD-1/PD-L1 inhibitors are currently in the first line therapy for lung cancers. Here we tested the expression of CMTM6 and PD-L1 in 81 lung cancers from 4 different histotypes. Among the 81 samples, 14 (17.3%) samples were negative of CMTM6, while the other 67 (82.7%) showed positive CMTM6 staining (Table 2). In contrast, for the PD-L1 expression, only 11 (13.6%) samples showed positive staining, while the other 70 (86.4%) samples were negative. Among the 81 samples, 65 samples were pre-diagnosed as NSCLC, among which only 2 (3%) were CMTM6 negative while 63 (97%) were CMTM6 positive. In contrast, among the other 16 SCLC samples, there were 12 (75%) CMTM6 negative and only 4 (25%) were positive. The expression level for CMTM6 in all 81 samples ranges from 14 cases of negative (0), 15 cases of weak (1), 39 cases of moderate (2) and 13 cases of strong (3), respectively. For PD-L1, all 11 positive staining were from NSCLC (16.9%), while none from SCLC. The typical CMTM6 and PD-L1 staining images from lung cancer samples are shown in Fig. 2.

### 3.3 Correlation of CMTM6 expression and clinical pathological parameters of lung cancers

**Fig. 3:**
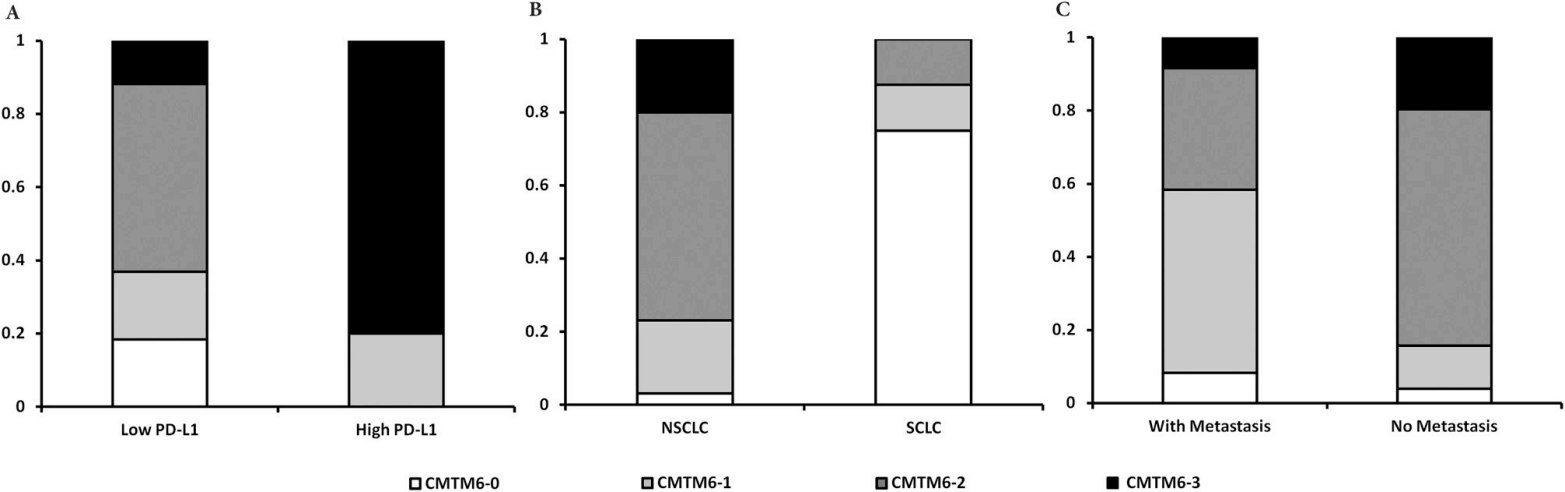
**CMTM6/PD-L1 expression correlation and association with lung cancer clinicalpathological parameters**. In a cohort of lung cancer patients' samples, correlation between CMTM6 and PD-L1 expression was analyzed and presented in A. Moreover, CMTM6 expression was analyzed against the clinical pathological parameters of these patients samples. B, CMTM6 expression is significantly different between SCLC and NSCLC(P = 0.001). C, CMTM6 expression is significantly different between patients with lymph node metastasis and patients without (P = 0.017).

The correlation between CMTM6 expression and lung cancer samples' clinicalpathological parameters were analyzed using the CMTM6 expression level (0–3) against patients' gender, age, histotypes and lymph nodes metastases. The result indicated that CMTM6 expression is correlated with lung cancers histotypes (higher CMTM6 in NSCLC than that in SCLC, P < 0.05) and inversely correlated with lymph nodes metastases (Chi-square test, P < 0.001), but not with patients’ age and gender (Fig. 3).

### 3.4 Correlation of CMTM6 expression and PD-L1 expression in lung cancers

The correlation between CMTM6 expression and PD-L1 expression were analyzed in this study. Strikingly, we observed that all 11 samples with PD-L1 positivity were with CMTM6 positive expression as well. Chi-square test showed that CMTM6 expression and PD-L1 expression were significantly correlated with P < 0.001. No samples were positive for PD-L1 expression if CMTM6 expression is not present (Table 3 and Fig. 3).

## 4 Discussion

Our result with TMA immunohistochemistry showed that CMTM6 expresses in 15 types of cancers, and this is consistent with previous report in which CMTM6 mRNA expression was detected from tumor samples of 30 different types of cancers by The Cancer Genome Atlas (TCGA) [6], indicating CMTM6 expression is common in most cancer types. For the other four negative tumor types, extended studies with big sample size are needed to further verify its expression in those cancer types and its relation with PD-L1 expression. Together with other 7 family members, CMTM6 belongs to the CKLF family protein that ubiquitously expressed in human tissues [8]. The function of CMTM6 was unknown until the latest publication reporting it as the important regulator for PD-L1 [6,7]. As one of the most important player in the immune checkpoint system, PD-L1 has been reported to be expressed in 7 types of cancers, including urothelial [9], lung [10], thyroid [11], cervical [12], skin [13], stomach [14] and melanoma [15], and membranous PD-L1 functions as a co-inhibitory factor in immune regulation [16]. In this study, we observed the PD-L1 positivity in 8 types of cancers, including bladder, cervical, liver, lung, melanoma, ovary, pancreatic, and stomach. This is basically consistent with the previous reports. Additional research on PD-L1 expression in pancreatic cancers is warranted as there is currently no effective therapy for pancreatic cancer [17].

We observed that all patient's samples with PD-L1 staining were positive for CMTM6, but it is not the case vice versa. This discovery suggests that, in different types of cancers, the expression of PD-L1 relies on the presence of CMTM6, which is consistent with the previous report in cellular model that PD-L1 relies on CMTM6 to efficiently carry out its inhibitory functions [7]. This result is important as gene expression regulation include CMTM6 expression are often more complicated than its expression at a single cellular level.

Lung cancer was chosen to be our focus of this study as PD-L1 associated immunotherapy is playing ever increasing role in lung cancer management. So far there are already five PD-1/PD-L1 inhibitor drugs approved by FDA, with four out of these 5 drugs are targeting NSCLC. To better understand the regulation of PD-L1 inhibitory impact on the immune systems, and design better therapeutical strategies, lung cancer is undoubtedly an ideal model for current study. A cohort of 81 lung cancers was included, among which 65 were NSCLC and 16 were SCLC. Our result indicated that PD-L1 is only positive in NSCLC (five adenocarcinomas and six squamous cell carcinomas), but not in any SCLC. This is consistent with the previous report [18]. For CMTM6, it is positive in most NSCLC (97%), while is positive in only 25% of SCLC. With all positive PD-L1 samples also expressing CMTM6, and stronger CMTM6 expression showing stronger PD-L1 expression (Fig. 2, Fig. 3), their expressions are significantly correlated. This is consistent with two previous independent studies reporting that CMTM6 functions as a critical regulator of PD-L1 [6,7]. It was reported that CMTM6 binding with PD-L1 could reduce PD-L1 ubiquitination and result in longer half life of PD-L1. Consequently, CMTM6 over-expression increases PD-L1 protein pool but not influences the PD-L1 transcription. Interference of CMTM6 impairs the PD-L1 expression in human tumor and dentritic cells [6]. In another report, supporting the idea from above paper, CMTM6 was shown to protect PD-L1 from being targeted by lysosome-mediated degradation and maintain PD-L1 expression on the cell surface. CMTM6 depletion, via the reduction of PD-L1, significantly alleviates the suppression of tumor-specific T cell activity *in vitro* and *in vivo* [7]. The correlation between CMTM6 and PD-L1 expression in NSCLC tissues observed in this study might be attributed to the mechanism described in the above two publications at the molecular level, but more importantly, revealed this important correlation in the clinical lung cancer samples. This is to date the first report of such a correlation in a cohort of lung cancer samples. Additionally, this study also for the first time demonstrated that CMTM6 expression is inversely correlated with lymph node metastasis. Future research is needed to confirm this relation in larger lung cancer samples and other cancer types. Equally important, a better understanding of this inverse correlation with prognosis and treatment outcome may be valuable for better lung cancer management.

The current study, for the first time, demonstrated that CMTM6 expression is correlated with lung cancer histotypes and inversely correlated with cancer metastases in clinical samples, supporting earlier reports in cellular models. We observed higher percentage of CMTM6 expression, together with PD-L1 expression, in NSCLC than SCLC, and in cancer without metastases than those with metastases. This can be explained by the different characteristics and the tumor microenvironment of these two types of cancers as indicated in previous report. NSCLC typically carries the genetic mutations with EGFR, ALK, BRAFV600E as well as ROS1. Compared with SCLC, NSCLC is epithelial cells origin and relatively less aggressive with the 5-year survival higher than 11–17%. It also relatively grows slower and metastasizes later than SCLC. As NSCLC and SCLC are encountering different tumor microenvironment and the slow growth of NSCLC may lead to longer exposure time of tumor cells to the immune system and activate the T cell function. To escape the immune surveillance, tumor cells will create/select advantageous mutations, including the high expression of CMTM6, to maintain the high level of PD-L1 and thus to suppress the cytotoxic T cell activation. In contrast, SCLC is more aggressive neoplasia arising from the neuroendocrine cells [18]. It usually grows fast and spread in early stage, with 5-year survival rate less than 7%. Frequently, it already grows beyond the lung when SCLC is diagnosed. SCLC carries the genetic mutation profile including ProGRP, NCAM, PGP9.5, and gastrin etc. Therefore, the different cell origin of NSCLC and SCLC, the individual genetic mutation profile, together with the different microenvironment may cause the higher expression of CMTM6/PD-L1 in NSCLC.

In Summary, we here reported that CMTM6 is expressed in diverse cancers and its expression is correlated with PD-L1 expression. In lung cancer, CMTM6 is correlated with NSCLC subtype and inversely correlated with metastases. The results from this study may serve to better understand the PD-L1 expression regulation in human cancer tissue, to better select immune response patients, and better design therapeutic strategies with CMTM6 as a combined or independent target.

## Disclosure/conflict of interest

There is no conflict of interest.
